# A neuroscientist’s guide to using murine brain atlases for efficient analysis and transparent reporting

**DOI:** 10.3389/fninf.2023.1154080

**Published:** 2023-03-09

**Authors:** Heidi Kleven, Ingrid Reiten, Camilla H. Blixhavn, Ulrike Schlegel, Martin Øvsthus, Eszter A. Papp, Maja A. Puchades, Jan G. Bjaalie, Trygve B. Leergaard, Ingvild E. Bjerke

**Affiliations:** Neural Systems Laboratory, Institute of Basic Medical Sciences, University of Oslo, Oslo, Norway

**Keywords:** brain atlases, FAIR data, reporting practices, spatial registration, rat brain, mouse brain, brain-wide analysis, neuroinformatics

## Abstract

Brain atlases are widely used in neuroscience as resources for conducting experimental studies, and for integrating, analyzing, and reporting data from animal models. A variety of atlases are available, and it may be challenging to find the optimal atlas for a given purpose and to perform efficient atlas-based data analyses. Comparing findings reported using different atlases is also not trivial, and represents a barrier to reproducible science. With this perspective article, we provide a guide to how mouse and rat brain atlases can be used for analyzing and reporting data in accordance with the FAIR principles that advocate for data to be findable, accessible, interoperable, and re-usable. We first introduce how atlases can be interpreted and used for navigating to brain locations, before discussing how they can be used for different analytic purposes, including spatial registration and data visualization. We provide guidance on how neuroscientists can compare data mapped to different atlases and ensure transparent reporting of findings. Finally, we summarize key considerations when choosing an atlas and give an outlook on the relevance of increased uptake of atlas-based tools and workflows for FAIR data sharing.

## Introduction

Converting the increasing amounts of multifaceted neuroscience data into knowledge about the healthy and diseased brain requires that relevant data are accumulated and combined in a common context. The FAIR principles set forward by Wilkinson et al. (2016), stating that data should be findable, accessible, interoperable, and re-useable, facilitate such data integration. Practical implementation of these principles in neuroscience can be achieved by using brain atlases as a common framework, equipping the data with metadata describing their location in the brain. Brain atlases contain standardized references to brain locations, and their utility for integrating neuroscience data is already well-established (Toga and Thompson, 2001; Zaslavsky et al., 2014; Bjerke et al., 2018b).

Neuroscientists use atlases at several stages of a research project, from planning and conducting studies to analyzing data and publishing results. A variety of atlases exist, revealing different features of rat and mouse (collectively referred to as murine) neuroanatomy. However, different atlases use various traditions for defining and naming brain regions, hampering interpretation, and comparison of data from locations specified using different atlases. Thus, while atlases provide common frameworks for neuroscience data integration, researchers might find it challenging to know which atlas to choose and how to use it. This makes it difficult for researchers to efficiently interpret and analyze their data using atlases, and for reporting and sharing data in accordance with the FAIR principles. Here, we provide a guide to using murine brain atlases for efficient analysis, reporting and comparison of data, offering the perspective that open volumetric brain atlases are essential for these purposes.

## Finding brain locations by navigating and interpreting atlases

**FIGURE 1:**
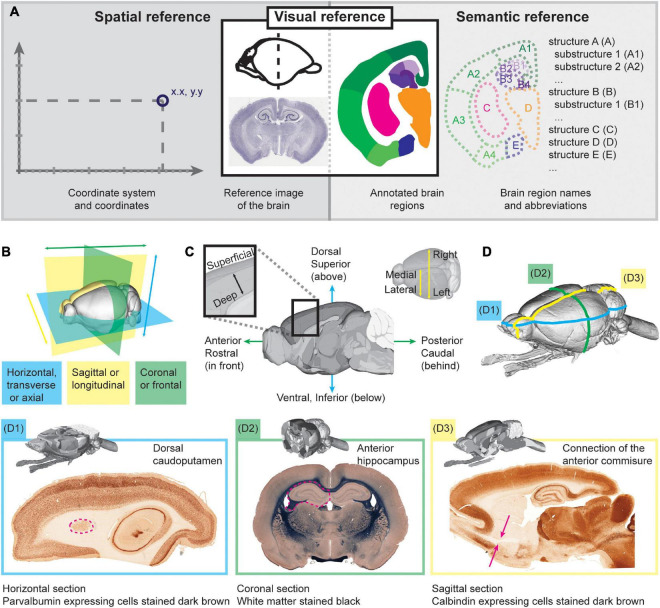
Navigating brain atlases to find anatomical locations. **(A)** Simplified version of the brain atlas ontology model (AtOM, Kleven et al., 2023a). The main elements of an atlas include the coordinate system, the reference image (here exemplified with a coronal platypus brain section; Mikula et al., 2007), the annotated brain regions, and the brain region names. The elements provide different entry points for navigating the atlas, through a spatial, semantic or visual reference. **(B)** Illustration of the three standard planes (horizontal, blue; sagittal, yellow; coronal, green) typically used to cut brain sections. **(C)** Illustration of the essential terminology typically used for indicating positions in the brain (e.g., the terms “rostral” and “caudal” to refer to positions towards the front and back of the brain, respectively). **(D)** Illustration of useful landmark regions in the murine brain [adapted from Bjerke et al. (2023)], with examples from the horizontal **(D1)**, coronal **(D2**; Leergaard et al., 2018), and sagittal **(D3)** planes.

There are two types of murine brain atlases: traditional two-dimensional (2D) atlases with serial section images (e.g., Paxinos and Watson, 2013; Swanson, 2018) and digital volumetric (3D) atlases (e.g., Papp et al., 2014; Barrière et al., 2019; Wang et al., 2020). The traditional atlases rank among the most cited neuroscience publications. However, they are limited by the distance between section images and the fixed plane(s) of orientation. They are also poorly suited for automated whole-brain analysis and digital workflows, and reuse of atlas images in publications may require permission from the publisher. The digital volumetric atlases are typically shared openly, and they allow data analysis independent of the plane of sectioning. The most detailed and commonly used volumetric atlas for the mouse is the Allen Mouse Brain Common Coordinate Framework (Allen Mouse Brain CCF; Wang et al., 2020), which has been instrumental for the acquisition and sharing of the Allen Institute’s large data collections (Lein et al., 2007; Oh et al., 2014; Tasic et al., 2016). For the rat, the most detailed volumetric atlas is the Waxholm Space atlas of the Sprague Dawley rat brain (WHS rat brain atlas; RRID:SCR_017124; Papp et al., 2014; Kjonigsen et al., 2015; Osen et al., 2019; Kleven et al., 2023a). Other murine brain atlases are also available [see summary by Barrière et al. (2019)]. Regardless of the 2D or 3D format, murine brain atlases can be navigated and interpreted using the spatial, visual, and semantic reference space (Figure 1A; Kleven et al., 2023a).

The *spatial reference* consists of a coordinate system and a reference image. The reference image of an atlas may originate from a single specimen (Papp et al., 2014) or represent a population average (Wang et al., 2020) of multiple specimens, with different brain region characteristics (e.g., cyto- or chemoarchitecture, and gene expression) visible depending on the modality. The reference image is made measurable through the coordinate system. Most brain atlases use a 3D Cartesian coordinate system with a defined origin and each of the x, y, z axes oriented in one of the standard anatomical planes. Atlases typically follow the neurological orientation of axes described by the right-anterior-superior (RAS) scheme, where the x-axis is oriented toward the right (R), the y toward anterior (A), and the z toward superior (S)^1^. The origin may be defined by skull features (stereotaxic coordinate system; Paxinos and Watson, 2013), internal landmarks (Waxholm Space; Papp et al., 2014), or the physical limits of the reference image such as the corner of a volume (Wang et al., 2020).

The *visual reference* consists of the reference image and a set of boundaries of brain regions (annotations), defined using criteria-based interpretations (e.g., differences in gene expression patterns, and changes in cyto-, myelo-, or chemoarchitecture). Easily recognizable features that are consistent across individuals are often used as landmarks when positioning an experimental image in an atlas (Sergejeva et al., 2015). For example, the beginning and end of easily distinguished brain regions, such as the caudoputamen or hippocampus (Figure 1D), are highly useful for orientation. Such landmarks are particularly useful for guiding and assessing the quality of the spatial registration of experimental section images to an atlas (Puchades et al., 2019; see section on analysis below), as well as for detecting abnormal anatomical features in the images. A selection of useful murine brain landmarks are given by Bjerke et al. (2023).

The *semantic reference* consists of the brain region annotations and their names. Regions, areas, and nuclei of the brain may be named after the person who first defined them, or after distinct features, such as their architecture or relative position within a broader region. While murine brain atlas terminologies often combine terms from different conventions, most atlases present white matter regions with a lower case first letter and gray matter regions with a capital first letter. Digital atlases may also use color coding schemes to indicate relationships between region annotations, e.g., using the same color for all white matter regions or for regions at the same level of the hierarchy of gray matter regions (Wang et al., 2020).

## Analyzing data using atlas-based tools and workflows

**FIGURE 2:**
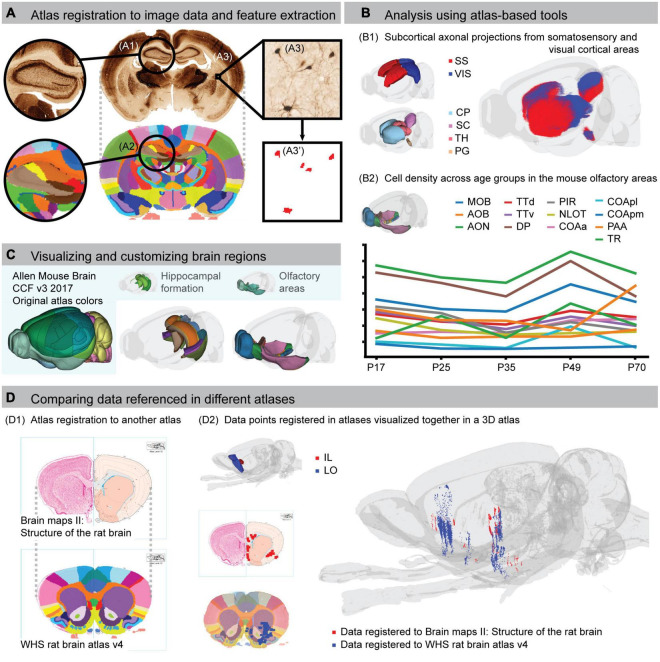
Using brain atlases for spatial registration, analysis, visualization and comparison of data. **(A)** Example of spatial registration of a histological section to the Waxholm Space (WHS) rat brain atlas. Landmark regions, such as the hippocampus **(A1,A2)**, are used to find corresponding positions between the image and the atlas. Features in the images **(A3)**, with spatial metadata from the registration, can be extracted **(A3’)**. The example in **(A)** shows a histological image stained for parvalbumin neurons registered to the WHS atlas. **(B)** The principal workflow of combining atlas registration with extracted features illustrated in **(A)** can be used for different types of atlas-based analyses. **(B1)** 3D dot map visualization of corticostriatal, corticotectal, and corticopontine axonal projections originating from the primary somatosensory cortex (SS, red) and visual cortex (VIS, blue) cortical areas, extracted from anterograde tract tracing data (Oh et al., 2014) registered to the Allen mouse brain CCFv3-2017 (Ovsthus et al., 2022). **(B2)** Analysis of dopamine 1 receptor positive cell densities in olfactory regions of the mouse brain across five postnatal day (P) age groups [y axis values not shown, preliminary data extracted from images provided by Bjerke et al. (2022)]. **(C)** Visualization of customized regions from the Allen mouse brain CCFv3-2017. The left panel shows the entire atlas with the default color scheme. The middle panel shows a transparent view of the brain with regions of the hippocampal formation color coded to their corresponding region in the WHS rat brain atlas, facilitating cross-species comparisons. In the right panel, to better visualize the extent of individual regions, they are coded with contrasting colors, whereas the original atlas uses the same or highly similar colors. **(D)** Example of how co-registration of brain atlases supports comparison of data referenced in different atlases. The stereotaxic atlas by Swanson (1998) has been spatially registered to the WHS rat brain atlas **(D1)**. Data that have been extracted and mapped to the two atlases can therefore be co-visualized in the same 3D space **(D2)**. In this example, the red points are extracted from a previous study where retrograde projections from injections in the infralimbic cortex were represented with schematic drawing of terminal fields onto atlas plates from the Swanson atlas (Figure 8, data mirrored for comparison; Hoover and Vertes, 2007). The blue points are extracted from a public dataset showing the anterograde projections originating from the lateral orbitofrontal cortex (case F1 BDA; Kondo et al., 2022). AOB, accessory olfactory bulb; AON, anterior olfactory nucleus; CP, caudoputamen; COAa, cortical amygdalar area, anterior part; COApl, cortical amygdalar area, posterior part, lateral zone; COApm, cortical amygdalar area, posterior part, medial zone; DP, dorsal peduncular area; IL, infralimbic cortex; LO, lateral orbitofrontal cortex; MOB, main olfactory bulb; NLOT, nucleus of the lateral olfactory tract; PAA, piriform-amygdalar area; PG, pontine gray; PIR, piriform area; SC, superior colliculus; SS, somatosensory area; TH, thalamus; TR, postpiriform transition area; TTd, taenia tecta dorsal part; TTv, taenia tecta ventral part; VIS, visual area.

Atlas coordinates provide spatial reference in machine-readable units. When coupled to the atlas terminology, they enable automated analysis of data registered to that atlas. A broad range of software incorporating atlases, here called atlas-based tools, are available to perform various digital analyses of brain image data. Atlas-based analyses rely on spatial registration, here defined as the process of assigning anatomical location to each pixel or voxel of the data (Figure 2A). This is achieved through aligning 2D and/or 3D data with the reference image of the atlas.

Several computational methods for registration of 2D image data to atlases have been developed. However, implementations are typically tailored to specific data types (e.g., fluorescent images or 3D data) and may require coding skills. Thus, tools with a graphical user interface that are applicable to a broad range of data types have also been developed, often incorporated as part of analytic workflows (Tappan et al., 2019; Ueda et al., 2020; BICCN Data Ecosystem Collaboration et al., 2022; Tyson and Margrie, 2022). An example of a standalone tool for spatial registration of histological sections to volumetric atlases is QuickNII (RRID:SCR_017978; Puchades et al., 2019). QuickNII is available with the WHS rat brain atlas (v2, v3, and v4) and the Allen Mouse Brain CCF (v3-2015 and v3-2017). Manual alignment of individual section images is relatively time-consuming, and can greatly benefit from a machine learning-based approach for section alignment, such as implemented in DeepSlice for coronal rat and mouse brain sections^2^ (Carey et al., 2022). While these tools rely on linear registration methods, murine brains show variability (Badea et al., 2007; Scholz et al., 2016) that cannot always be compensated for by using linear transformations. Histological brain sections are also prone to physical damage and deformities caused by tissue processing (Simmons and Swanson, 2009). To amend this, non-linear adaptations of linearly registered murine images can be achieved using VisuAlign (RRID:SCR_017978).

Murine brain research increasingly includes 3D imaging data acquired by magnetic resonance or diffusion tensor imaging (Gesnik et al., 2017), serial two-photon imaging (Oh et al., 2014) or light sheet microscopy (Ueda et al., 2020). As these data are spatially coherent and avoid the deformities and damage seen in histological sections, they lend themselves well to volume-to-volume registration with 3D reference atlases. Several groups have developed computational methods for this type of alignment [see review by BICCN Data Ecosystem Collaboration et al. (2022)^3^ and Tyson and Margrie (2022)], most often toward the Allen Mouse Brain CCF. The Elastix toolbox (Klein et al., 2010) also offers a collection of algorithms that can be used for 3D image registration.

Spatially registered image data can be used in analytic workflows for region-based annotation, quantification, and reconstruction of features in and across images. Such workflows typically entail three steps: (1) registration of image data (2D or 3D) to an atlas, (2) feature extraction, and (3) quantification and/or visualization of extracted features (Figure 2B). Several authors have demonstrated how such workflows can be used to quantify features of the brain (Kim et al., 2017; Pallast et al., 2019; Newmaster et al., 2020). Although many use custom code, workflows based on both commercial and open source tools exist. For example, NeuroInfo from MBF Bioscience (Tappan et al., 2019) supports reconstruction of sections into a volume and registration to an atlas with automatic image segmentation and quantification. Alternatively, the free and open source QUINT workflow (Yates et al., 2019) aligns histological section images to atlas, and applies the same alignment to segmented images where a given feature (e.g., labeled cell bodies) is represented with a single color using the Nutil tool (Groeneboom et al., 2020, RRID:SCR_017183). For registration of electrode positions or viral expression, the HERBS software (Fuglstad et al., 2023) offers integrated spatial registration and feature extraction, where results can be directly visualized in 3D.

## Visualization of atlases and image data

Spatial metadata makes it possible to view and interact with atlases and image data in several online atlas viewers. The Scalable Brain Atlas Composer^4^ (SBA; Bakker et al., 2015) is capable of viewing 2D or 3D images of a range of different formats. In addition, the SBA can view spatial metadata (e.g., from QuickNII or DeepSlice) together with .png images of histological sections. Another online tool is the EBRAINS interactive atlas viewer^5^, which is available for all versions of the WHS rat brain atlas and the Allen Mouse Brain CCF. This viewer also allows upload of user-defined data. For example, the user can drag-and-drop a .nii volume to view it in the three standard planes and slice it in arbitrary angles, with region annotations available as an overlay. Additionally, 3D rendering of coordinate-based data such as point clouds representing tracer distributions or cell bodies can be achieved online via MeshView (RRID:SCR_017222). MeshView allows slicing of volumes containing point clouds in user-defined planes for inspection and analysis of topographical patterns (see e.g., Tocco et al., 2022).

## Customizing brain atlases for analysis and visualization

Open access digital brain atlases allow researchers to customize the anatomical annotations, reference images, or terminology in the atlas for specific analyses. Several tools have taken advantage of this, and enable the user to customize the atlas in an interactive way through a user interface. For example, QCAlign (RRID:SCR_023088; Gurdon et al., in preparation) allows interactive exploration of the hierarchy and grouping of brain region names that can subsequently be used in the QUINT workflow to merge brain regions into broader, custom regions for analysis. This may for example be used to merge and rename regions to make them compatible with a different naming convention, e.g., to enable cross-species comparison where atlases for different species must be harmonized (Figure 2C; Bjerke et al., 2021). Merging regions can also facilitate teaching by introducing students to macrostructure before revealing details. A more advanced use case is to modify or create new brain region annotations. For this purpose, the open access segmentation software like e.g., ITK-SNAP (Yushkevich et al., 2006) is useful for viewing and editing volumetric files across a range of different formats.

## Comparing atlases and data mapped to different atlases

A major challenge across atlases is the variety of brain region annotations and terminologies (Swanson, 2000; Bohland et al., 2009). When different names are used to refer to the same brain region, or when similar names are used for partly overlapping ones, confusion is inevitable (Van De Werd and Uylings, 2014; Bjerke et al., 2020). Unequivocal referencing (see “Citing atlases and anatomical locations”) can mitigate some of this, but the challenge remains that different terminologies often reflect differences in criteria for annotating brain regions. Differences in the brain region annotations across atlases and their versions make it difficult to compare data where locations are reported using different atlases. To amend this, Khan et al. (2018) performed a co-registration between versions of the stereotaxic rat brain atlases. They migrated data originally registered to one of Paxinos and Watson’s (1986) earliest atlases to its corresponding plate in Swanson’s (2018) most recent versions, making the data comparable. It is also possible to migrate legacy data to a volumetric atlas, upon which different datasets can be compared and co-visualized in 3D space (Figure 2D). To support such efforts, we have spatially registered several versions of the traditional stereotaxic atlases to the WHS rat brain atlas and Allen Mouse Brain CCF (Bjerke et al., 2020). The co-registration data are available for download through the EBRAINS Knowledge Graph^6^ in QuickNII compatible format (see, e.g., Bjerke et al., 2018b and the related EBRAINS project on the web portal)^7^. The open access Swanson atlases are also available in an interactive viewer. Thus, the variety of atlases available and the fact that different data will be referenced using different atlases, while a challenge, can be mitigated by mapping atlases to each other.

## Citing atlases and anatomical locations

Brain locations may be specified by names or coordinates, but to be reproducible a specific citation of the atlas used is required. A challenge is that researchers often report the name of a brain region that they are familiar with, and not the name recorded in the brain atlas they have used (Bjerke et al., 2020). For example, a researcher may use “striatum” to refer to the dorsal part of the striatal complex called “caudoputamen” in most atlases. While the researcher may see these names as interchangeable, a reader may consider “striatum” to include the nucleus accumbens, which is also a common convention. This creates a source of confusion even when citing an atlas. We have previously put forward a set of recommendations to unambiguously refer to anatomical locations in the murine brain (Bjerke et al., 2018a), e.g., highlighting the importance of using terms as they appear in the atlas, or otherwise specifying how the terms used relate to those in the atlas.

Citation of an atlas should include the version. This is easy with traditional atlases following a linear versioning track (Paxinos and Watson, 2007; Swanson, 2018). However, volumetric digital atlases are often provided with several files that may be versioned separately. To facilitate correct citation, Kleven et al. (2023a) proposed an Atlas ontology model and an overview of the versioning of the two most commonly used volumetric murine brain atlases, the WHS rat brain atlas and the Allen Mouse Brain CCF. Beyond consistent and correct citation of atlases, any customizations (see “Customizing brain atlases for analysis and visualization”) should be clearly documented (Rodarie et al., 2021).

When using atlas-based software, it is important to be aware that software versioning is often independent of the atlas versioning. Thus, the software and atlas versions will have separate citation policies (usually along with separate RRIDs; Bandrowski and Martone, 2016), and should be named and cited accordingly when reporting data acquired using atlas-based software. Multiple atlases may be available in the same tool, in which case it is critical to record which atlas and version was used.

## How to choose a brain atlas?

With several atlases available, it is challenging to know what sets different atlases apart and choosing the most appropriate brain atlas depends on its intended purpose. First, reproducibility and availability should be considered. In most laboratories, there are 2D book atlases on the shelf. While the mere physical availability of book atlases makes them convenient to use during experimental work, many of them are challenging to use for transparent reporting due to restrictive licenses and high costs for reproducing figures. Choosing an open access atlas makes it easier to communicate findings transparently and ensure their replicability. Second, reference images differ among atlases and should ideally match the experimental data at hand. For example, different strains are used across available rat brain atlases, with Wistar used in the Paxinos atlases (Paxinos and Watson, 2013) and Sprague Dawley used in Swanson’s atlases (Swanson, 2018) and in the Waxholm Space rat brain atlas (Papp et al., 2014). Other characteristics, such as age category, sex, wild type or transgenic specimens, and data modality will also influence how well the atlas can be applied to experimental data. In general, the more characteristics match between the subjects used in an experiment and the reference atlas, the better the atlas will represent the data, an essential consideration for analyses. A third important feature of an atlas is its interoperability with other atlases and related analysis software. A researcher intending to analyze data based on an atlas will benefit from a digital 3D atlas incorporated in digital tools and workflows. Whether the atlas has been used in a similar study or is part of a data integration effort may also be relevant (Oh et al., 2014; Bjerke et al., 2018b; Erö et al., 2018), as this will facilitate comparison of findings with published data and enable similar comparisons in the future.

## The evolution of brain atlases

Brain atlases are continuously created and refined to reflect researchers’ needs for appropriate references for subjects of different ages (developmental or aging) or strains, or for data acquired with various imaging modalities, to mention some. In particular, there has been an increasing focus on the need for a common coordinate framework to map data across different developmental stages. A challenge with these resources is that they either do not cover early postnatal and embryonic stages (Newmaster et al., 2020), or have delineations that are not readily compatible with adult atlases (Young et al., 2021). Additionally, there is need for brain atlases capturing the fine details of brain regions distinguished by e.g., topographical organization of connections (Zingg et al., 2014; Hintiryan et al., 2016). For example, Chon et al. (2019) created an atlas with highly granular annotations of the mouse caudoputamen by using cortico- and thalamo-striatal connectivity data. By combining delineations from Allen Mouse Brain CCF and the Franklin and Paxinos atlases, this atlas also helps alleviating some of the inconsistencies in nomenclature (Chon et al., 2019). As these examples show, several atlases are required to cater to current needs, and future methodologies and findings will add further possibilities and needs for continued development and refinement of atlases. For such new atlases to enable researchers to cite, (re-)analyze, and compare data independently of the original atlas used, it is essential that they are openly shared and properly documented (Kleven et al., 2023a).

## Conclusion and outlook: Open atlases help make data FAIR

In this perspective, we have provided a guide to murine brain atlases with a focus on how to use them for spatial registration, efficient analysis, and transparent reporting of data. Powerful analytic pipelines will hopefully incentivize more researchers to spatially register their data to atlases. We anticipate that the increasing availability and automation of atlas-based software with graphical user interfaces will fundamentally change how neuroscience will be performed in the future and lead to a major increase in the amount of more easily interpretable neuroscience data. For the field to benefit maximally from this shift, it is crucial that datasets and spatial metadata are openly shared in a public repository. This can be achieved with open access volumetric atlases as essential resources for making the wealth of multifaceted neuroscience data FAIR.

## Data availability statement

The original contributions presented in this study are included in the article/supplementary material, further inquiries can be directed to the corresponding author.

## Author contributions

HK and IB conceptualized and wrote the manuscript with input from TL. HK, IR, MØ, and IB prepared figures. All authors contributed to the development of concepts and resources described in the manuscript, and to manuscript revision.

## Footnotes

1
https://nipy.org/nibabel/neuro_radio_conventions.html

2
www.deepslice.org

3
https://www.biorxiv.org/content/10.1101/2022.10.26.513573v1

4
https://scalablebrainatlas.incf.org/composer/

5
https://interactive-viewer.apps.hbp.eu

6
https://search.kg.ebrains.eu/

7
https://search.kg.ebrains.eu/?category=Dataset&q=swanson#e2a1f65d-41fa-4bb1-ba48-93b36174a405
